# Incidence and predictive risk factors of postoperative sepsis in orthopedic trauma patients

**DOI:** 10.1007/s10195-016-0437-4

**Published:** 2016-11-15

**Authors:** Nikita Lakomkin, Vasanth Sathiyakumar, Brandon Wick, Michelle S. Shen, A. Alex Jahangir, Hassan Mir, William T. Obremskey, Ashley C. Dodd, Manish K. Sethi

**Affiliations:** The Vanderbilt Orthopaedic Institute Center for Health Policy, 1215 21st Avenue South, Suite 4200, Medical Center East, South Tower, Nashville, TN 37232 USA

**Keywords:** Septicemia, Septic shock, Complication, Adverse event, NSQIP, Orthopedics

## Abstract

**Background:**

Postoperative sepsis is associated with high mortality and the national costs of septicemia exceed those of any other diagnosis. While numerous studies in the basic orthopedic science literature suggest that traumatic injuries facilitate the development of sepsis, it is currently unclear whether orthopedic trauma patients are at increased risk. The purpose of this study was thus to assess the incidence of sepsis and determine the risk factors that significantly predicted septicemia following orthopedic trauma surgery.

**Materials and methods:**

56,336 orthopedic trauma patients treated between 2006 and 2013 were identified in the ACS-NSQIP database. Documentation of postoperative sepsis/septic shock, demographics, surgical variables, and preoperative comorbidities was collected. Chi-squared analyses were used to assess differences in the rates of sepsis between trauma and nontrauma groups. Binary multivariable regressions identified risk factors that significantly predicted the development of postoperative septicemia in orthopedic trauma patients.

**Results:**

There was a significant difference in the overall rates of both sepsis and septic shock between orthopedic trauma (1.6%) and nontrauma (0.5%) patients (*p* < 0.001). For orthopedic trauma patients, ventilator use (OR = 15.1, *p* = 0.002), history of pain at rest (OR = 2.8, *p* = 0.036), and prior sepsis (OR = 2.6, *p* < 0.001) were significantly associated with septicemia. Statistically predictive, modifiable comorbidities included hypertension (OR = 2.1, *p* = 0.003) and the use of corticosteroids (OR = 2.1, *p* = 0.016).

**Conclusions:**

There is a significantly greater incidence of postoperative sepsis in the trauma cohort. Clinicians should be aware of these predictive characteristics, may seek to counsel at-risk patients, and should consider addressing modifiable risk factors such as hypertension and corticosteroid use preoperatively.

*Level of evidence* Level III.

## Introduction

The development of postoperative sepsis is a common yet serious complication that has been associated with significant morbidity and death, resulting in mortality rates ranging from 28 to 50% [1–4]. Its prevalence has been documented by numerous studies in the general medical as well as orthopedic literature, with postoperative rates of septicemia doubling between 1997 and 2006 [5–9]. The severity and frequency of this condition have had a profound effect on inpatient care. Between 2000 and 2008, the rate of sepsis-induced hospitalizations more than doubled, and the development of septicemia has been associated with significant increases in inpatient length of stay and resource utilization [1, 4].

These challenges have created remarkable implications for healthcare expenditure in the United States. National costs associated with hospitalizations due to sepsis amounted to 16.7 billion dollars in 2001 [10], and had surpassed 20 billion dollars by 2011, thereby making sepsis the single most expensive condition treated that year [11]. In an effort to reduce the total cost of care in an increasingly challenging fiscal landscape, emerging reimbursement models are beginning to shift the costs associated with perioperative complications and readmissions onto providers [12–14]. Although numerous basic science studies in orthopedics have suggested that orthopedic trauma injuries may be associated with immune responses that facilitate sepsis [15–18], it is currently unclear whether orthopedic trauma patients are at increased risk for developing sepsis or septic shock. Existing studies have correlated trauma to large groups of adverse events, making it difficult to extrapolate associations to septic complications [19–22]. However, the relationship between sepsis and high mortality, its enormous financial costs, and the increasing emphasis on quality metrics make it imperative that orthopedic surgeons are able to identify trauma patients who may be more likely to develop the condition. Early identification and supplemental monitoring of patients with an increased propensity for sepsis may aid traumatologists in maintaining lower overall complication rates, thereby improving clinical outcomes in high-risk groups.

Despite this, there is a dearth of literature assessing the preoperative risk factors that are associated with septicemia. Existing studies have examined broad diagnostic categories that are not specific to orthopedic surgery and do not achieve consensus regarding predictive characteristics [5, 6, 23]. Furthermore, the relationship between orthopedic trauma and sepsis following orthopedic intervention remains unexplored in the current literature. Thus, the purpose of this study was to utilize the American College of Surgeons National Surgical Quality Improvement (NSQIP) database in order to elucidate whether trauma patients are at increased risk for developing sepsis and septic shock compared to orthopedic patients without traumatic injuries. In addition, the demographic and preoperative characteristics that served as significant predictors of sepsis were identified using multivariable regression analysis. Early identification of patients who may be at increased risk for developing sepsis may facilitate the timely modification of controllable risk factors and potentially improve clinical outcomes.

## Materials and methods

### Study design and NSQIP characteristics

This investigation utilized the NSQIP database, the characteristics of which have been described in a number of previous studies exploring postoperative outcomes [24–26]. Data collection occurs prospectively from over 250 medical centers throughout the country, and selection bias is mitigated by systematic sampling and auditing procedures, resulting in <1.6% disagreement regarding collected variables [27, 28]. The utility of the NSQIP database in perioperative quality improvement in orthopedic surgery has been well documented [29]. Because this study used data from a de-identified national database rather than human participants, IRB approval was not required.

### Data collection

Patients who underwent orthopedic surgery procedures between 2006 and 2013 were identified by applying 1066 unique current procedural terminology (CPT) codes to the NSQIP database. Among these, an additional 89 trauma-specific CPT codes were utilized in order to identify those who underwent orthopedic trauma procedures. This distinction was employed in order to create two cohorts of “trauma” and “nontrauma” patients.

Demographic and select preoperative characteristics including age, race, gender, functional dependence status, body mass index (BMI), and American Society of Anesthesiologists (ASA) classification were collected for all patients in the trauma and nontrauma subgroups. In addition, preoperative comorbidities incorporating smoking status, presence of diabetes, ventilator use, history of chronic obstructive pulmonary disorder (COPD), congestive heart failure (CHF), disseminated cancer, ≥10% weight loss in the six months preceding surgery, bleeding disorders, prior myocardial infarction (MI), hypertension (HTN), ascites, varices, pneumonia, peripheral vascular disease (PVD), pain at rest, altered sensation, dialysis, renal failure, coma, quadriplegia, hemiplegia, paraplegia, transient ischemic attack (TIA), cerebrovascular accident (CVA), CNS tumors, transfusions, dyspnea, radiation therapy, angina, chemotherapy, and sepsis were recorded. Surgical characteristics included the type of anesthesia used (general or other), presence of orthopedic trauma, do not resuscitate (DNR) status, and whether or not the case was emergent.

Patients were considered to have developed septic complications if they (1) demonstrated clinical signs of systemic inflammatory response syndrome (SIRS) as well as associated blood culture (sepsis) or (2) presented with symptoms of SIRS along with blood culture and documented organ dysfunction (septic shock) within 30 days following surgery.

### Statistical analysis

Selected demographic characteristics and preoperative comorbidities were compared between the trauma and nontrauma cohorts using chi-squared analysis or Fisher’s exact tests, as appropriate. Similarly, chi-squared analyses were employed in order to assess differences in the rates of postoperative septicemia between the orthopedic trauma and nontrauma subgroups.

Patient demographics, preoperative comorbidities, and surgical characteristics for the entire cohort were explored for potential associations with the development of postoperative sepsis using bivariate chi-squared analysis. Factors discovered to be significantly associated with sepsis were subsequently evaluated in a binary multivariable regression model in order to identify significant predictors of septic complications. This was performed for all orthopedic surgery patients in order to determine whether or not orthopedic trauma was a risk factor for postoperative sepsis. Patients with incomplete data for any risk factor were excluded from the analysis.

The above analysis was replicated for solely the orthopedic trauma subgroup, thereby identifying the risk factors significantly associated with the development of septicemia for trauma patients while controlling for all other characteristics and comorbidities. Tolerance values for each variable were calculated in order to discover potential multicollinearity between the risk factors. All comparative tests were two-tailed and statistical significance was set at *p* < 0.05. All statistical analyses were performed using IBM SPSS Statistics 22 (SPSS Inc., Armonk, NY, USA).

## Results

### Trauma vs. nontrauma demographics

A total of 361,402 orthopedic surgery patients were identified in the 2005–2013 NSQIP database. Of these, 56,336 (15.6%) underwent orthopedic trauma procedures. A comparison of patient demographics between the two cohorts (trauma and nontrauma) is presented in Table 1. There were significant differences (*p* < 0.001) between orthopedic trauma and nontrauma patients with respect to all of the collected demographics. Orthopedic trauma patients were more likely to be female (*n* = 36,360; 64.6%), over the age of 65 (*n* = 34,576; 61.4%), and functionally dependent (*n* = 928; 13.4%). Meanwhile, a larger proportion of nontrauma patients had a BMI of greater than 35 (*n* = 70,573; 23.4%).

**Table 1:** Comparison of selected characteristics between orthopedic trauma and nontrauma patients

Demographics	Orthopedic trauma (*N* = 56,336)	Nontrauma (*N* = 305,066)	*p*
Age (mean ± SD)			
≥65	34,576 (61.4%)	124,743 (40.9%)	<0.001
<65	21,762 (38.6%)	180,317 (59.1%)	
Gender			
Male	19,939 (35.4%)	142,880 (46.9%)	<0.001
Female	36,360 (64.6%)	161,829 (53.1%)	
Diabetes	9031 (16%)	41,866 (13.7%)	<0.001
Functionally dependent	928 (13.4%)	983 (2.5%)	<0.001
BMI			
≥35	5561 (10.4%)	70,573 (23.4%)	<0.001
<35	47,661 (89.6%)	234,185 (76.6%)	
ASA score			
>2	33,053 (58.8%)	113,704 (37.3%)	<0.001
≤2	23,203 (41.2%)	190,864 (62.7%)	

### Trauma vs. nontrauma incidence of sepsis

**Fig. 1:**
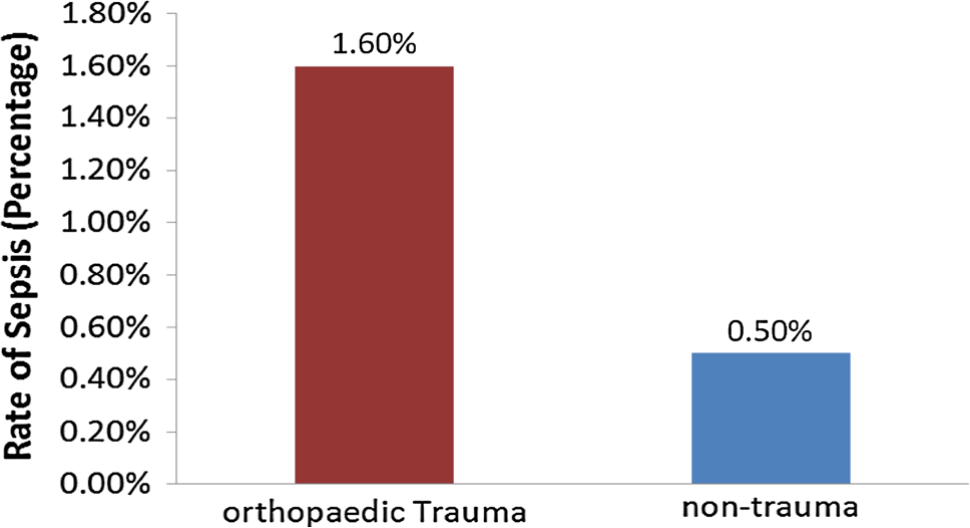
Comparison of the rates of septicemia following orthopedic trauma and nontrauma intervention

A total of 2524 patients (0.7%) developed postoperative sepsis or septic shock. There was a significant difference in the overall rate of sepsis-related complications between orthopedic trauma (1.6%, 916/56,336) and nontrauma (0.5%, 1608/305,068) patients (*p* < 0.001, Fig. 1). Similar differences were found between the two cohorts in regard to the incidence of sepsis (1.1 and 0.4%, respectively, *p* < 0.001) and septic shock (0.6 and 0.1%, respectively, *p* < 0.001).

### Significant predictors of postoperative sepsis

Bivariate chi-squared analyses exploring the relationship between all of the demographic, preoperative, and surgical characteristics and postoperative septicemia revealed significant associations for all characteristics except varices, angina, and pregnancy.

In a binary, multivariable regression analysis incorporating patient factors that were significantly associated with sepsis for all orthopedic patients, orthopedic trauma was not found to be a significant predictor of the development of postoperative septic complications (*p* > 0.05). The results of the multivariable regression for all orthopedic procedures are presented in Table 2.

**Table 2:** Predictors of postoperative sepsis for all orthopedic surgery patients

Risk factor	Odds ratio	95% confidence interval	*p*
Demographics			
African American race	1.58	1.13–2.21	0.008
Male gender	1.41	1.13–1.76	0.002
Age >65	1.38	1.06–1.78	0.015
Preoperative risk factors			
Ascites	4.72	1.24–17.9	0.023
Transfusion of >4 units of PRBC	4.31	1.42–13.1	0.01
Use of ventilator	4.29	1.49–12.31	0.007
History of sepsis	2.69	1.96–3.70	<0.001
Functional dependence	2.21	1.57–3.09	<0.001
Corticosteroid use	2.04	1.39–3.00	<0.001
ASA score >2	1.9	1.42–2.55	<0.001
Hypertension	1.43	1.10–1.87	0.008
Orthopedic trauma	1.17	0.89–1.53	0.263

The bivariate chi-squared analyses for all of the factors pertaining to the trauma cohort revealed significant associations (*p* < 0.05) between postoperative sepsis and all of the collected characteristics except for race, anesthesia type, smoking status, varices, angina, CNS tumor, quadriplegia, and pregnancy. The remaining (significant) factors were incorporated into a binary multivariable regression model for trauma patients.

The demographic and preoperative characteristics that statistically predicted the development of postoperative septicemia for orthopedic trauma patients are depicted in Table 3. Patients who were male, required the use of a ventilator, had taken corticosteroids, presented with hypertension, had an ASA score of >2, experienced pain at rest, were functionally dependent, or had a history of sepsis were significantly more likely to develop septic complications (*p* < 0.001–*p* = 0.046). Hypertension (OR = 2.1, *p* = 0.003) and the use of corticosteroids (OR = 2.1, *p* = 0.016) were the modifiable comorbidities that were predictive of the development of postoperative sepsis and septic shock. The computed tolerance values for all of the examined variables exceeded 0.10, thus demonstrating no multicollinearity between risk factors.

**Table 3:** Significant predictors of postoperative sepsis for orthopedic trauma patients

Risk factor	Odds ratio	95% confidence interval	*p*
Demographics			
Male gender	1.5	1.0–2.2	0.046
Preoperative risk factors			
Use of ventilator	15.1	2.8–81.5	0.002
Pain at rest	2.8	1.1–7.2	0.036
History of sepsis	2.6	1.7–4.0	<0.001
Functional dependence	2.3	1.4–3.7	0.001
Hypertension	2.1	1.3–3.3	0.003
Corticosteroid use	2.1	1.2–4.0	0.016
ASA score >2	2.1	1.1–3.7	0.016

*ASA* American Society of Anesthesiologists, *BMI* body mass index, *PRBC* packed red blood cells

## Discussion

The development of postoperative sepsis is a very serious complication that has been associated with high morbidity, mortality, and increased length of inpatient stay. In 2011, national healthcare payments for septicemia exceeded those of any other diagnosis, and numerous basic science studies have suggested that orthopedic trauma may be associated with immunosuppression that contributes to sepsis [16, 17]. Despite this, very little data describing the rates of postoperative sepsis in general orthopedics and orthopedic trauma exist in the current literature. While orthopedic trauma patients have been shown to develop greater rates of adverse events, no study has examined septic complications in isolation from other less serious conditions. As such, the purpose of this study was to assess differences in the rates of postoperative sepsis between trauma and nontrauma cohorts, evaluate orthopedic trauma as a potential predictor of postoperative sepsis in orthopedic surgery, and identify the preoperative comorbidities that significantly predicted the incidence of septic complications following orthopedic trauma surgery. Our data derived from a prospective, multicenter database demonstrated that rates of postoperative sepsis differed significantly between trauma and nontrauma subgroups. In addition, this study identified eight preoperative characteristics that were significant risk factors for septicemia in trauma patients. Hypertension and the use of corticosteroids were the modifiable comorbidities that were predictive of the development of sepsis following orthopedic trauma.

The overall rate of septic complications for the entire cohort of orthopedic patients was 0.70%, representing a very slight increase in incidence from the 0.68% determined using 2005–2011 NSQIP data [30]. Orthopedic trauma patients experienced postoperative septicemia at a rate of 1.6%. This is consistent with previous studies assessing the incidence of sepsis in other surgical populations, which reported rates of between 0.9 and 2.3% [6, 31, 32].

Bivariate analysis demonstrated that orthopedic trauma patients develop postoperative sepsis at significantly greater rates than those undergoing nontraumatic procedures. To the authors’ knowledge, this is the first study to assess the incidence of postoperative septicemia for a large cohort of orthopedic trauma patients. While Sathiyakumar et al. demonstrated that orthopedic trauma [21] and hip fracture patients [20] tend to experience adverse events at greater rates, sepsis was examined as part of a cohort that included many other complications, rather than in isolation. Similarly, other studies examining adverse events following orthopedic trauma do so using large clusters of complications, making it difficult to evaluate postoperative septicemia following these procedures. Our finding that orthopedic trauma patients are more likely to develop sepsis is consistent with a number of basic science studies purporting that traumatic injury induces an immunosuppressive effect on the rest of the body, thus facilitating septicemia [33–35]. In evaluating differences in interleukin-10 release between patients undergoing reamed and unreamed femoral nailing, Smith et al. concluded that reamed intramedullary nailing was associated with decreased immune response [16]. Their findings were subsequently corroborated by Giannoudis et al., who documented a significant anti-inflammatory response following major orthopedic trauma [17]. This delayed hypersensitivity has since been demonstrated to increase patients’ risk of developing sepsis following trauma [36, 37]. As such, our clinical findings for a large cohort of orthopedic trauma and nontrauma patients are in line with the associations and suggestions described in the orthopedic basic science literature.

Although orthopaedic trauma status was significantly associated with postoperative sepsis for all orthopedic surgery patients using bivariate chi-squared analysis, it interestingly fell out of significance when employing a binary multivariable regression model. Because of the low standard error and high tolerance associated with this variable, it is unlikely that significant multicollinearity between patient factors impacted the results. Further studies are thus needed in order to elucidate the predictive effect of trauma on the incidence of sepsis in the context of other preoperative variables.

In order to better understand the parameters that were associated with the greater incidence of septicemia among orthopedic trauma patients, preoperative risk factors that significantly predicted septic complications were identified. Several of these predictors are consistent with the existing literature examining the risk factors for sepsis, although no prior studies have done so for orthopedic trauma. In particular, associations between male gender [23, 38, 39], age greater than 65 [40], and sepsis-related complications have been reported in a number of studies. In their analysis of the risk factors of sepsis after multiple trauma, Wafaisade et al. utilized a multivariate regression to identify male gender, increased age, and pre-existing conditions as significant predictors [23]. However, their cohort comprised an array of trauma patients, only a fraction of whom experienced orthopedic injuries. Additionally, individual preoperative comorbidities were not evaluated, making it difficult to identify potential avenues for clinical change. Preoperative mechanical ventilation, which was reported as a risk factor for sepsis in this study, has also been associated with increased risk of postoperative sepsis in pediatric cardiothoracic surgery [41]. To our knowledge, this is one of the very few studies to report hypertension, prior pain at rest, history of sepsis, and corticosteroid use as predictive risk factors for septicemia, and the first to do so for orthopedic surgery.

Although several of our identified risk factors are demographic in nature and cannot be modified prior to surgery, they nevertheless form the groundwork on which risk calculations and further analyses can be made. Awareness of factors such as age, functional dependence, history of pain at rest, and prior sepsis could facilitate additional monitoring of these patients in conjunction with internists, and is important for truly informed consent. Clinicians may consider counseling patients who present with prior history of sepsis along with other predictive risk factors.

Preoperative hypertension and use of corticosteroids, however, are modifiable factors that warrant further research exploring their relationship with septic complications. Hypertension, for instance, has previously been associated with increased mortality from sepsis [42, 43]. Hypertensive patients may also be sicker overall and have previously been documented to experience a higher number of complications compared to their nonhypertensive counterparts in orthopedic cases [44–47]. It is therefore possible that achieving preoperative control over high blood pressure may yield clinical benefits for surgery-bound patients. The use of corticosteroids prior to surgery may also be clinically relevant for orthopedic trauma patients. Exposure to corticosteroids has been associated with not only a greater risk of developing postoperative infection [48–50], but the potential to induce immunosuppression and adrenocortical suppression during sepsis [51]. The known immunosuppressive effect of corticosteroid use coupled with the demonstrated role of immunosuppression in the development of sepsis following orthopedic trauma necessitates further studies examining this topic [17, 37, 52]. While it is plausible that eliminating corticosteroids could mitigate this factor, further evidence is necessary to draw meaningful conclusions and make sound clinical recommendations.

Preoperative mechanical ventilation has previously been associated with increased incidence of sepsis in children, and prolonged ventilator use was reported to correlate with incidence of postoperative infections [41, 53]. Although preoperative modification of this risk factor may not be possible in a sizeable number of cases, clinicians could consider weaning patients off ventilators prior to surgery. Further research examining the association between mechanical ventilation and postoperative infections in orthopedics is needed.

The use of NSQIP-derived risk factors as part of a screening algorithm has already been demonstrated to reduce sepsis-associated morbidity and mortality. In a study by Moore et al., the authors utilized the NSQIP database to identify age, presence of comorbidities, and emergency surgery as risk factors that were predictive of sepsis in the general surgery population [31]. Their subsequent implementation of mandatory screening for these high-risk groups resulted in reduced mortality due to septicemia at their affiliated medical center. The strength of our study lies in the identification of specific comorbidities as well as demographics that could be incorporated into such a screening model. Further investigations assessing how increased monitoring of patients with the risk factors identified in the present study may affect incidence of sepsis in orthopedic trauma are thus warranted.

The results of our study should be evaluated in the context of limitations that were inherent to utilizing a national database. First, the development of sepsis or septic shock was recorded within the first 30 days following the procedure. It is possible that additional episodes of sepsis-related complications occurred after that time frame, resulting in our reported rates of sepsis underestimating actual occurrence. Second, the NSQIP dataset incorporates only 20% of clinical volume from reporting medical centers. However, a stringent auditing and sampling technique is employed in order to mitigate selection bias, and the validity of the NSQIP dataset for these kinds of prognostic investigations has been shown [24, 28]. Third, the study was confined to using the variables collected and stored in the prospective database. Miscellaneous preoperative characteristics including patients’ socioeconomic status as well as orthopedic-specific postoperative complications such as hardware failure could not be evaluated. Nevertheless, the large, prospective, and multicenter origins of the data coupled with the stringent clinical evidence required for sepsis classification mitigates potential bias and is a strength compared to prior studies. In addition, the wide variety of preoperative comorbidities that were collected and controlled for in the multivariable regression mitigated potential confounding factors while yielding several predictors that were corroborated in previous studies.

This investigation utilized more than 50,000 orthopedic trauma patients from over 250 medical centers throughout the country. The data demonstrated an increased incidence of postoperative septicemia among patients undergoing orthopedic trauma procedures compared to those with nontraumatic orthopedic intervention. In addition, several unique predictive factors, including hypertension, corticosteroid use, ventilator use, and history of pain at rest, were identified. Clinicians may be interested in counseling at-risk groups and consider addressing modifiable comorbidities before surgery.

## References

[1] Hall MJ, Williams SN, DeFrances CJ (2011). Inpatient care for septicemia or sepsis: a challenge for patients and hospitals. NCHS Data Brief.

[2] Hund E (2001). Neurological complications of sepsis: critical illness polyneuropathy and myopathy. J Neurol.

[3] Brun-Buisson C, Doyon F, Carlet J et al (1995) Incidence, risk factors, and outcome of severe sepsis and septic shock in adults. French ICU Group for Severe Sepsis. JAMA 274(12):968–974 7674528

[4] Daniels R (2011). Surviving the first hours in sepsis: getting the basics right (an intensivist’s perspective). J Antimicrob Chemother.

[5] Mokart D, Leone M, Sannini A (2005). Predictive perioperative factors for developing severe sepsis after major surgery. Br J Anaesth.

[6] Bateman BT, Schmidt U, Berman MF (2010). Temporal trends in the epidemiology of severe postoperative sepsis after elective surgery: a large, nationwide sample. Anesthesiology.

[7] Vogel TR, Dombrovskiy VY, Carson JL (2010). Postoperative sepsis in the United States. Ann Surg.

[8] Malina RM (2010). Early sport specialization: roots, effectiveness, risks. Curr Sports Med Rep.

[9] Fitzgerald RH, Nolan DR, Ilstrup DM (1977). Deep wound sepsis following total hip arthroplasty. J Bone Joint Surg Am.

[10] Angus DC, Linde-Zwirble WT, Lidicker J (2001). Epidemiology of severe sepsis in the United States: analysis of incidence, outcome, and associated costs of care. Crit Care Med.

[11] Torio CM, Andrews RM (2006, 2011) National inpatient hospital costs: the most expensive conditions by payer: statistical brief #160. In: Healthcare Cost and Utilization Project (HCUP) statistical briefs. Agency for Health Care Policy and Research, Rockville

[12] Mechanic RE (2011) Opportunities and challenges for episode-based payment. N Engl J Med 365(9):777–779 10.1056/NEJMp1105963 21864162

[13] Hemmila MR, Jakubus JL, Maggio PM (2008). Real money: complications and hospital costs in trauma patients. Surgery.

[14] Chen C, Ackerly D (2014) Beyond ACOs and bundled payments: Medicare’s shift toward accountability in fee-for-service. JAMA 311(7):673–674 10.1001/jama.2014.11 24549543

[15] Wanner GA, Keel M, Steckholzer U (2000). Relationship between procalcitonin plasma levels and severity of injury, sepsis, organ failure, and mortality in injured patients. Crit Care Med.

[16] Smith RM, Giannoudis PV, Bellamy MC (2000). Interleukin-10 release and monocyte human leukocyte antigen-DR expression during femoral nailing. Clin Orthop.

[17] Giannoudis PV, Smith RM, Perry SL (2000). Immediate IL-10 expression following major orthopaedic trauma: relationship to anti-inflammatory response and subsequent development of sepsis. Intensive Care Med.

[18] Giannoudis PV, Smith RM, Banks RE (1998). Stimulation of inflammatory markers after blunt trauma. Br J Surg.

[19] Pape H-C, Griensven MV, Hildebrand FF (2008). Systemic inflammatory response after extremity or truncal fracture operations. J Trauma.

[20] Sathiyakumar V, Greenberg SE, Molina CS (2015). Hip fractures are risky business: an analysis of the NSQIP data. Injury.

[21] Sathiyakumar V, Thakore RV, Greenberg SE (2015). Adverse events in orthopaedics: is trauma more risky? An analysis of the NSQIP data. J Orthop Trauma.

[22] Lakomkin N, Greenberg SE, Obremskey WT, et al. (2015) The risk of adverse events in orthopaedic trauma varies by anatomic region of surgery: an analysis of fifty thousand four hundred and twenty one patients. Int Orthop 39(11):2153–2159. 10.1007/s00264-015-2899-z 26183144

[23] Wafaisade A, Lefering R, Bouillon B (2011). Epidemiology and risk factors of sepsis after multiple trauma: an analysis of 29,829 patients from the Trauma Registry of the German Society for Trauma Surgery. Crit Care Med.

[24] Belmont PJ, Goodman GP, Kusnezov NA (2014). Postoperative myocardial infarction and cardiac arrest following primary total knee and hip arthroplasty: rates, risk factors, and time of occurrence. J Bone Joint Surg Am.

[25] Schoenfeld AJ, Ochoa LM, Bader JO et al (2011) Risk factors for immediate postoperative complications and mortality following spine surgery: a study of 3475 patients from the National Surgical Quality Improvement Program. J Bone Jt Surg 93(17):1577–1582 10.2106/JBJS.J.01048 21915571

[26] Schoenfeld AJ, Carey PA, Cleveland AW (2013). Patient factors, comorbidities, and surgical characteristics that increase mortality and complication risk after spinal arthrodesis: a prognostic study based on 5,887 patients. Spine J Off J N Am Spine Soc.

[27] Shiloach M, Frencher SK, Steeger JE (2010). Toward robust information: data quality and inter-rater reliability in the American College of Surgeons National Surgical Quality Improvement Program. J Am Coll Surg.

[28] Ingraham AM, Richards KE, Hall BL (2010). Quality improvement in surgery: the American College of Surgeons National Surgical Quality Improvement Program approach. Adv Surg.

[29] Schilling PL, Hallstrom BR, Birkmeyer JD (2010). Prioritizing perioperative quality improvement in orthopaedic surgery. J Bone Joint Surg Am.

[30] Molina CS, Thakore RV, Blumer A et al (2015) Use of the National Surgical Quality Improvement Program in orthopaedic surgery. Clin Orthop 473(5):1574–1581 10.1007/s11999-014-3597-7 PMC4385340 24706043

[31] Moore LJ, Moore FA, Todd SR et al (2010) Sepsis in general surgery: the 2005–2007 National Surgical Quality Improvement Program perspective. Arch Surg Chic 145(7):695–700 10.1001/archsurg.2010.107 20644134

[32] Osborn TM, Tracy JK, Dunne JR (2004). Epidemiology of sepsis in patients with traumatic injury. Crit Care Med.

[33] Ertel W, Keel M, Bonaccio M (1995). Release of anti-inflammatory mediators after mechanical trauma correlates with severity of injury and clinical outcome. J Trauma.

[34] Tan LR, Waxman K, Scannell G (1993). Trauma causes early release of soluble receptors for tumor necrosis factor. J Trauma.

[35] Roberts CS, Pape H-C, Jones AL (2005). Damage control orthopaedics: evolving concepts in the treatment of patients who have sustained orthopaedic trauma. Instr Course Lect.

[36] Meakins JL, Pietsch JB, Bubenick O (1977). Delayed hypersensitivity: indicator of acquired failure of host defenses in sepsis and trauma. Ann Surg.

[37] Giannoudis PV (2003). Current concepts of the inflammatory response after major trauma: an update. Injury.

[38] Aube H, Milan C, Blettery B (1992). Risk factors for septic shock in the early management of bacteremia. Am J Med.

[39] Martin GS, Mannino DM, Eaton S (2003). The epidemiology of sepsis in the United States from 1979 through 2000. N Engl J Med.

[40] Martin GS, Mannino DM, Moss M (2006). The effect of age on the development and outcome of adult sepsis. Crit Care Med.

[41] Simsic JM, Kanter KR, Kirshbom PM (2007). Does preoperative mechanical ventilation affect outcomes in neonates undergoing cardiac surgery?. Cardiol Young.

[42] Kercher J, Xerogeanes J, Tannenbaum A (2009). Anterior cruciate ligament reconstruction in the skeletally immature: an anatomical study utilizing 3-dimensional magnetic resonance imaging reconstructions. J Pediatr Orthop.

[43] Sibbald WJ (1978) Pulmonary hypertension in sepsis: measurement by the pulmonary arterial diastolic-pulmonary wedge pressure gradient and the influence of passive and active factors. Chest 73(5):583 10.1378/chest.73.5.583 648208

[44] Yang S, Nguyen ND, Center JR, Eisman JA, Nguyen TV (2014). Association between hypertension and fragility fracture: a longitudinal study. Osteoporos Int.

[45] Henderson CY, Ryan JP (2015). Predicting mortality following hip fracture: an analysis of comorbidities and complications. Ir J Med Sci.

[46] Jiang JJ, Phillips CS, Levitz SP, Benson LS (2014). Risk factors for complications following open reduction internal fixation of distal radius fractures. J Hand Surg Am.

[47] Harstedt M, Rogmark C, Sutton R, Melander O, Fedorowski A (2015). Impact of comorbidity on 6-month hospital readmission and mortality after hip fracture surgery. Injury.

[48] Mastropietro CW, Barrett R, Davalos MC (2013). Cumulative corticosteroid exposure and infection risk after complex pediatric cardiac surgery. Ann Thorac Surg.

[49] Aberra FN, Lewis JD, Hass D (2003). Corticosteroids and immunomodulators: postoperative infectious complication risk in inflammatory bowel disease patients. Gastroenterology.

[50] Merkler AE, Saini V, Kamel H (2014). Preoperative steroid use and the risk of infectious complications after neurosurgery. Neurohospitalist.

[51] Sprung CL, Brezis M, Goodman S (2011). Corticosteroid therapy for patients in septic shock: some progress in a difficult decision. Crit Care Med.

[52] Polk HC (1987). Non-specific host defence stimulation in the reduction of surgical infection in man. Br J Surg.

[53] Toufektzian L, Patris V, Sepsas E et al (2015) Does postoperative mechanical ventilation predispose to bronchopleural fistula formation in patients undergoing pneumonectomy? Interact Cardiovasc Thorac Surg 10.1093/icvts/ivv149 26069338

